# Immune-mediated muscle wasting and cell survival - the tale of a curious gene

**DOI:** 10.1186/1479-5876-9-S2-I8

**Published:** 2011-11-23

**Authors:** James A Ross

**Affiliations:** 1 Tissue Injury & Repair Group, University of Edinburgh, MRC Centre for Regenerative Medicine, Edinburgh, UK

## Background

Cancer cachexia remains a major clinical problem with the majority of advanced cancer patients suffering from aspects of this complex syndrome. Two key features of cancer cachexia include anorexia and an acute phase response, both of which contribute directly to accelerated wasting. Some of the metabolic abnormalities in cancer cachexia are similar to those involved in trauma and sepsis and involve a systemic inflammatory response with profound alterations in liver metabolism, together with loss of lean body mass. Such depletion of lean tissue (principally skeletal muscle) is an important factor contributing to the decreased survival of cancer patients. Skeletal muscle is the main labile source of amino acids in the body and this muscle is broken down to provide amino acids to the liver for the continuing, relentless acute phase response which is a feature of cancer cachexia.

A novel 24kDa, heavily glycosylated, cachectic factor which can trigger muscle proteolysis during the process of cancer cachexia was identified first from a cachexia-inducing mouse tumor [1] and from the urine of patients with pancreatic carcinoma and weight-loss [2]. The factor was identified as being a product of the human cachexia associated protein (HCAP) gene [3] and was later referred to as proteolysis inducing factor (PIF) which had a small core peptide (PIF-CP) of less than 3kDa and was extensively glycosylated. This glycosylated factor was shown by us to drive the acute phase response and to induce an inflammatory response from human hepatocytes [4] and other cell types [5,6].

The story then became more complex. From work in an unrelated area, a peptide was identified as a neuronal survival peptide (YP-30) in rat neuronal cells subjected to oxidative stress [7] and this peptide proved to be identical in sequence to the PIF-CP. The rat gene encoding the YP-30 peptide was cloned and named the DSEP gene [8]. Around the same time, and again in a completely unrelated area, a group in Tubingen, working on skin antibiotics, identified and cloned the dermcidin (DCD) gene [9] which produced the dermcidin antibiotic peptide DCD-1. The HCAP, DSEP and DCD genes are completely homologous. In a bizarre twist of biological fate this gene encodes different products derived from separate regions of the gene. One product is the 30 amino acid core peptide of proteolysis inducing factor (PIF-CP), which is identical to the YP-30 neuronal survival peptide, and an entirely separate product, the dermcidin antibiotic peptide DCD-1. In a further, unrelated area, the DCD gene was identified as a candidate oncogene in invasive breast cancer [10]. We later went on to demonstrate that the DCD gene encoding PIF-CP, YP-30 and DCD-1 could protect various tumour cells from oxidative stress and hypoxia [11,12] and that the portion of the gene responsible for this protection was that encoding the PIF-CP/YP-30 peptide [13]. Expression of the gene also appeared to confer a proliferative effect on cells.

## Aims

The aim of this paper is to describe the biology of the dermcidin gene and to identify the portion of the gene responsible for inducing cell proliferation.

## Results and conclusion

In this study, we confirm a proliferative effect of DCD overexpression in the HuH7 human hepatic cell line. The proliferation is abrogated by prevention of PIF-CP translation or inactivation of its calcineurin-like phosphatase domain by site-directed mutagenesis. Prevention of translation of the DCD-1 antibiotic peptide had no effect. Treatment of cells with a 30 amino acid synthetic PIF-CP induced an increase in proliferation. Pathway analysis revealed several gene networks involved in the cellular response to the peptide, one with VEGFB as a hub and two other networks converging on Fos and Myc.
